# Pursuing use of optimal formulations for paediatric HIV epidemic control – a look at the use of LPV/r oral pellets and oral granules

**DOI:** 10.1002/jia2.25267

**Published:** 2019-04-15

**Authors:** Christine Y Malati, Rachel Golin, Lisa O'Brien, Nandita Sugandhi, Meena Srivastava, Chris Larson, Benjamin R Phelps

**Affiliations:** ^1^ United States Agency for International Development Washington DC USA; ^2^ Independent Researcher Toronto Canada; ^3^ ICAP at Columbia New York NY USA; ^4^ United Parcel Service Columbus OH USA

**Keywords:** lopinavir/ritonavir, HIV infections, children, infants, protease inhibitor, antiretroviral therapy, optimal regimens

## Abstract

**Introduction:**

Despite a significant reduction in mother‐to‐child transmission of HIV, an estimated 180,000 children were infected with HIV in 2017, and only 52% of children under 15 years of age living with HIV (CLHIV) are on life‐saving antiretroviral therapy (ART). Without effective treatment, half of CLHIV die before the age of two years and only one in five survives to five years of age.

**Discussion:**

Over the past four years, the United States Food and Drug Administration tentatively approved new formulations of lopinavir/ritonavir (LPV/r) in the form of oral pellets and oral granules. However, the slow uptake of the aforementioned formulations in the low‐ and middle‐income countries with the highest paediatric HIV burden is largely due to three challenges: limited manufacturing capacity; current unit cost of the pellets and granules; and slow uptake of these new formulations by policy makers and health care workers.

**Conclusions:**

Solutions to overcome these barriers include ensuring availability of an adequate supply of LPV/r oral pellets and oral granules, considering all programmatic and clinical factors when selecting paediatric ART formulations, and leveraging current resources to decrease paediatric HIV morbidity and mortality.

## 1 Introduction

Despite a significant reduction in mother‐to‐child transmission of HIV (MTCT), an estimated 180,000 children were infected with HIV in 2017, and only 52% (44% to 73%) of children under 15 years of age living with HIV (CLHIV) are on life‐saving antiretroviral therapy (ART) 1.

Among CLHIV cohorts receiving ART, many demonstrate unacceptably low viral suppression 2, 3. The historical use of single‐dose nevirapine (sdNVP) in prevention of mother‐to‐child transmission (PMTCT) programmes has led to drug resistance and treatment failure among children receiving NVP‐based ART regimens. Currently, pretreatment drug resistance rates in children range from 3% to almost 70% 4, 5. Nonnucleoside reverse transcriptase inhibitor (NNRTI) resistance contributes significantly to treatment failure among CLHIV, with children on NVP‐based regimens twice as likely to have HIV drug resistance than those on protease inhibitors (PIs) such as lopinavir/ritonavir (LPV/r) 6. The HIV treatment community must leverage current opportunities to improve paediatric morbidity and mortality, for without effective treatment, half of CLHIV die before the age of two years and only one in five survive to five years of age 7, 8, 9.

According to studies in adults, integrase strand transfer inhibitor (INSTI) therapy is better tolerated than PI therapy, and has higher efficacy compared to NVP. The global HIV community is advocating for an ongoing transition to new integrase inhibitor‐based ART regimens including dolutegravir (DTG) and raltegravir (RAL);tenofovir 300 mg/lamivudine 300 mg/dolutegravir 50 mg fixed‐dose combination cannot be used in patients less than 30 kg 9. The WHO Optimal Paediatric ARV Formulary states that a DTG‐based regimen may be recommended as a preferred first‐line ART regimen, including infants and children with approved DTG dosing. However, based on the WHO, the current DTG formulation of a 50 mg tablet can be used in CLHIV who are ≥6 years of age and who weigh ≥20 kg, with approval for lower age and weight bands expected in late 2019 9, 10, 11, 12. Moreover, although RAL is the preferred first‐line ARV for neonates and one of the alternative first‐line ARVs for children, paediatric HIV treatment programmes have slowly introduced RAL, largely due to limited manufacturing capacity by the innovator company and due to the cost of RAL compared to NVP and LPV/r. However, use of RAL for neonates and children would allow patients to remain on an INSTI for the entire duration on ART, as they transition to DTG once they reach the appropriate weight 9. Fortunately, Merck recently announced new access prices for RAL formulations, which should improve the availability of these formulations (Merck, private communication, Dec 2018). Implementation studies are required to study the administration by caregivers of RAL granules for suspension as it requires more than ten steps for reconstitution 13. Until DTG and RAL become readily accessible for infants and young children, LPV/r‐based regimens remain the only available optimal first‐line ART for infants and young children.

LPV/r, currently available as oral solution, heat‐stable tablets, oral pellets and oral granules, can be used to construct a more robust ART regimen compared to a regimen containing NVP 14. Protease inhibitors, including LPV/r, are generally more robust than NNRTIs in efficacy, and therefore are essential when NNRTI‐resistance is expected (such as in infants and children whose mothers have received PMTCT regimens) or when treatment failure on an NNRTI‐regimen is confirmed 14, 15. While, the use of LPV/r oral solution is critical for patients that are less than three months of age due to the risk of aspiration with LPV/r oral granules and LPV/r oral pellets, the LPV/r oral solution formulation has challenges for distribution in resource limited settings, where cold chain requirements and poor palatability constrain uptake and adherence 11, 15. Moreover, cutting, crushing, chewing or dissolving the heat‐stable tablet LPV/r formulation has an unpredictable impact on pharmacokinetics, making it challenging to ensure a therapeutic dose is administered to infants and children 16, 17.

In May 2015, the United States Food and Drug Administration (US FDA) tentatively approved a new formulation of LPV/r: oral pellets 18. This formulation (lopinavir 40 mg/ritonavir 10 mg; manufactured by Cipla Limited) is packaged in a capsule that can be easily opened and sprinkled over soft food or, in the case of a young infant, placed in expressed breast milk or infant formula 19, 20. In August 2018, US FDA tentatively approved a similar formulation of LPV/r (40 mg/10 mg) in the form of oral granules, manufactured by Mylan 21. LPV/r oral pellets and oral granules offer many advantages over oral solution and heat‐stable tablets because of their ease of administration across multiple dosing ranges, ability to be stored at ambient temperature, and increased palatability due to taste‐masking by the milk or semi‐solid food co‐administered with the pellets. A transition to LPV/r oral pellets or oral granules provides an important intermediate formulation until infants reach an age and weight when they can transition to intact, heat‐stable tablets. According to the memorandum issued in January 2019, the Antiretroviral Procurement Working Group has stated that LPV/r oral pellets and oral granules are clinically equivalent and the dosing frequency is equivalent; however, the majority of the implementation studies have been conducted in lopinavir/ritonavir pellets 22.^1^

## 2 Discussion

### 2.1 Barriers to roll‐out of LPV/r oral pellet

Despite proven efficacy, tolerability and several comparative advantages, the uptake of this newer LPV/r formulation has been significantly lower than expected 23, 24. This section reflects on the lesson learned with regards to implementation of the oral pellets due to the increased amount of experience with the oral pellets compared to oral granules. This slow uptake in low‐ and middle‐income countries with the highest paediatric HIV burden is largely due to three challenges: 1 limited manufacturing capacity of the sole supplier; 2 current unit cost of the oral pellets; and 3 slow uptake of the new formulation by policy makers and health care workers (HCWs).

#### 2.1.1 Limited manufacturing

The paediatric ARV market is complex, due largely to the small number of patients eligible for paediatric formulations. With the success of PMTCT programmes, the population in need of paediatric ARVs will continue to decline, creating an additional risk to the market. As multiple international organizations and donors collaborate to introduce LPV/r oral pellets into routine clinical paediatric treatment, supply has become a challenge. Currently, Cipla is the sole manufacturer approved to manufacture LPV/r oral pellets, and Mylan is the sole manufacturer approved to manufacture LPV/r oral granules. The limited number of suppliers remains a fundamental limitation to the pace of LPV/r oral pellet and oral granule formulation roll‐out.

The current estimated global demand for LPV/r oral pellets and oral granules is difficult to ascertain. Due to the limited availability of LPV/r oral pellets and LPV/r oral granules, countries are reluctant to transition patients from NVP based regimens to LPV/r based regimens. Although Cipla recently received an approval from the US FDA for a modification that will yield an increase in manufacturing capacity, such increase remains unable to meet existing UNAIDS paediatric HIV estimates and epidemic control treatment targets. According to UNAIDS, in 2017, 1.8 million children under the age of 15 years are infected with HIV. If we estimate that, fifty percent of these children and young adolescents weigh between 3 and 13.9 kg, then 729,000 packs of oral pellets are required on a monthly basis to reach UNAID's second “90” treatment coverage. This is greater than ten times the current monthly manufacturing capacity Cipla is able to manufacture of LPV/r oral pellets. Unfortunately, the ongoing manufacturing constraints, as experienced through stock outs in Kenya and Uganda, may erode confidence in adequate supply and limit the transition to this optimal formulation.

Furthermore, due to the differences in administration of oral pellets and oral granules, it is not recommended to substitute between oral pellets and oral granules. Based on feedback from civil society in Uganda, the use of oral pellets AND oral granules within a country could create confusion among HCWs and caregivers.

#### 2.1.2 Current unit cost considerations

While advantages of the LPV/r oral pellet formulation have been clearly described, the unit cost is currently greater than that of LPV/r oral solution. Despite this difference, three supply chain cost advantages of the pellet formulation help offset its higher per‐unit price – lower weight, less storage cost and less wastage. Oral pellets weigh one‐third as much as the oral solution, significantly lowering shipping costs. Specifically, the cost to ship a one year supply of LPV/r oral pellets is 62% less than the cost to ship LPV/r oral solution. (See Table 1). Moreover, oral pellets only require a quarter of the storage space compared to LPV/r oral solution, and do not require refrigeration or cold‐chain packaging. As a result, the cost to store a one year supply of LPV/r oral pellets is 70% less than the cost to store LPV/r oral solution. The pellet formulation is also much more resilient, leading to less product wastage. LPV/r oral solution which requires 2°C to 8°C cold chain handling may quickly be rendered ineffective simply due to inconsistent refrigeration between the manufacturer and consumption by the patient. Many limited resource, high HIV burden settings may be prone to equipment malfunction and electricity outages and any interruption in the cold chain may negatively impact the pharmacokinetics, resulting in a substandard, wasted product. Furthermore, the cost of shipping and storage of LPV/r oral granules is approximately 50% greater than the cost of shipping and storage of LPV/r oral pellets.

**Table 1:** Total landed cost of select paediatric ART regimens^a,b^ 29, 30

Complete regimen	Total landed cost for 1000 patients for
Weight band: 3.0 to 5.9 kg	**Two months**
LPVr oral solution + ABC/3TC	$92,894
LPVr oral pellets + ABC/3TC	$53,086
LPVr oral granules + ABC/3TC	$51,186
Weight band: 6.0 to 9.9 kg	**One year**
LPVr oral solution + ABC/3TC	$884,355
LPVr oral pellets + ABC/3TC	$487,014
LPVr oral granules + ABC/3TC	$468,964
Weight band: 10.0 to 13.9 kg	**One year**
LPVr oral pellets + ABC/3TC	$1139,173
LPVr oral granules + ABC/3TC	$1092,623
LPVr heat stable tablets + ABC/3TC	$572,475
EFV + ABC/3TC	$146,218
Weight band: 14.0 to 19.9 kg	**One year**
LPVr oral pellets + ABC/3TC	$790,428
LPVr oral granules + ABC/3TC	$760,978
LPVr heat stable tablets + ABC/3TC	$320,990
EFV + ABC/3TC	$186,107
Weight band: 20.0 to 24.9 kg	**One year**
DTG + ABC/3TC	$225,892
EFV + ABC/3TC	$212,699
Weight band: 25.0 to 29.9 kg	**One year**
DTG + ABC/3TC	$183,605

ART, antiretroviral therapy; BID, twice daily; Caps, capsules; LPV/r, lopinavir/ritonavir; QAM, every morning; QPM, every evening; RAL, raltegravir.

^a^The grand total includes the cost for dosing a patient with consideration to the largest packaging required, the shipping cost and the storage cost with or without refrigeration as appropriate rounded to the nearest dollar. Costs are associated with USG funded procurement, shipping and distribution. Procurements that are procured with non‐USG funds will incur higher costs; ^b^additional ten bottles added for possible spills and re‐dosing due to poor palatability.

At the patient level, LPV/r oral solution is poorly tolerated and often spit out or regurgitated by infants due to its poor taste. Infants may ingest product that has been properly refrigerated; however if infants are ingesting substandard amounts of LPV/oral solution, they may acquire LPV/r resistance and consequently such patients would require a more expensive second‐ or third‐line regimen. Although the unit cost of oral pellets and oral granules is higher than that of oral solution or oral tablets, the direct and indirect cost savings associated with use of LPV/r oral pellets, as summarized above, are significant 25.

#### 2.1.3 Slow uptake by HCWs and caregivers

HCWs and caregivers are often slow to adopt unique new formulations 24. The unique formulation of oral pellets and oral granules requires both groups to engage with a previously uncommon method of administration and this may result in decreased acceptance and uptake 19. Since the oral pellets and oral granules cannot be crushed or dissolved in liquid, effective prescribing requires additional time educating the caregiver. Without such guidance, caregivers may have difficulty opening the capsules or may fail to administer oral pellets with age‐appropriate solids and oral solutions to achieve therapeutic dosing. The practical challenges of administration of oral pellets and oral granules in conjunction with breastfeeding, or assisting a child who refuses oral pellets or oral granules, must also be addressed.

Moreover, sequencing of formulations becomes complex as providers seek to follow treatment guidelines while using the most palatable formulation for each age and weight bracket. Though LPV/r oral pellet palatability is promising compared to liquid formulation in young infants, CHAPAS‐2 II study findings from Uganda suggest that, among older children, the LPV/r tablet formulation can be more palatable than oral pellets 23. Age‐related transitions between three different ART formulations used in young infants that are exposed for the purpose of ART (NVP solution, LPV/r oral solution, LPV/r oral pellets or LPV/r oral granules) offer additional complexity that may influence uptake among HCWs 26.

### 2.2 Addressing the Barriers to Oral Pellets and Oral Granules

In light of the context highlighted above, the authors recommend the following to accelerate the appropriate transition to LPV/r pellets, and to maximize treatment outcomes:

#### 2.2.1 Recommendation 1: Ensure there is adequate LPV/r oral pellet or LPV/r oral granule supply

Given the current limited supply of oral pellets, it is critical to perform appropriate forecasting to ensure product availability can be confirmed with the manufacturer of the respective product 23, 24. The current supply constraints are expected to improve over the next twelve months as the current manufacturer increases capacity and as the capacity of Mylan to manufacture LPV/r oral granule increases, and thus, now is the time to start planning. As Ministries of Health work to create an implementation plan, important quantification assumptions include the estimated MTCT rate, the number of pre‐ART CLHIV <3 years of age who are to be initiated on LPV/r, the number of CLHIV <3 years old who are currently on a suboptimal or failing ART regimen (e.g. NVP‐based regimens), and the number of eligible CLHIV who can successfully transition to LPV/r 100 mg/25 mg tablets. (Once children reach 10.0 kg in weight and can safely swallow a fully intact LPV/r 100 mg/25 mg tablet, they can be switched from pellets to this less expensive option.) It is especially important to consider use of LPV/r oral solution for lower weight bands, and reserve the use of LPV/r oral pellets and LPV/r oral granules for use during weight bands such as 6.0 to 9.9 kg and 10.0 to 13.9 kg, and potentially 14.0 to 19.9 kg. Ensuring availability of all types of LPV/r formulations will ensure consistent access to treatment and potentially limit the use of more costly second‐ and third‐line regimens for paediatric HIV treatment.

The product life cycle of LPV/r oral pellets and the product life cycle of LPV/r oral granules is dependent upon the timing of the introduction of dolutegravir containing regimens for CLHIV. The anticipated aggregated manufacturing capacity of LPV/r oral pellets and oral granules can only address nearly fifty percent of the anticipated need for paediatric treatment based on UNAIDS targets. Therefore, careful planning with the use of LPV/r oral solution in concert with oral pellets or in concert with oral granules is paramount. Furthermore, the need for all paediatric ARVs for the indication of treatment will continue to decline as children grow and require adult formulations and as the incidence rate of newborn infections will continue to decline. While access to LPV/r oral pellets and oral granules remains a critical priority in the short and medium terms, the use of protease inhibitors for children will be replaced with the use of integrase strand transfer inhibitors in the years ahead.

#### 2.2.2 Recommendation 2: When analysing cost, consider all contributors

As outlined above, the cost of oral pellets is not only driven by unit costs. Shipping and supply chain savings are essential considerations when calculating the cost of the transition. Oral pellets and oral granules offer an opportunity to ensure infants and young children have uninterrupted access to optimal child‐friendly formulations while avoiding unnecessary, avoidable wastage of suboptimal formulations. Through its improved palatability, use of LPV/r oral pellets and oral granules is likely to lead to higher rate of treatment tolerability and adherence, resulting in greater treatment efficacy with a more durable infant and young child ART regimen, and therefore improved clinical outcomes.

Management of multiple formulations within a supply chain carries an added cost due to the need for forecasting, storage and distribution costs associated with each product. If patients are successfully treated with age‐appropriate, child‐friendly LPV/r formulations, national HIV programmes could streamline procurement of optimal ARVs and potentially avoid the need for costly second‐ and third‐line ART regimens for CLHIV who are less than 3 years of age.

#### 2.2.3 Recommendation 3: Streamline introduction of new paediatric ART regimens and formulations

By the end of 2017, some high HIV burden countries, including Kenya and Uganda introduced LPV/r oral pellets into their national HIV programmes. This introduction was facilitated by the development of information, education and communication materials for policy makers, HCWs and caregivers 27. The ongoing LIVING study, conducted by Drugs for Neglected Diseases Initiative (DNDi), is assessing the transition to LPV/r oral pellets in Uganda and Kenya 28. As practical, front‐line issues such as HCW, caregiver and child acceptability become better understood, implementation will continue to improve. Best practices identified and lessons learned during the introduction of LPV/r oral pellets can inform introduction of future paediatric ART formulations, including integrase inhibitors. With the anticipated arrival of new paediatric ARV formulations and ART regimens (e.g. the DTG 10 mg scored tablet and new fixed‐dose combinations such as ABC/3TC/LPV/r and ABC/3TC/DTG), there is a need to concurrently develop strategies to simplify and standardize administration across regimens and formulations to help accelerate uptake of optimal paediatric HIV treatment 29.

## 3 Conclusion

It is essential to proactively pursue advanced, effective paediatric ART first‐line regimens to achieve HIV epidemic control and reduce HIV–associated morbidities. With careful consideration of the factors outlined above, efficacious, child‐friendly first‐line ARV formulations such as LPV/r oral pellets and oral granules have the potential to prolong and save thousands of young lives, if implemented in a coordinated fashion such that demand does not exceed supply. Lessons learned from the roll‐out of these newer formulations can be leveraged during implementation of new paediatric ARV formulations.

## Competing Interest

The authors declare that they have no competing interests.

## Authors’ contributions

C.M., L.O. and R.G. conceived and outlined paper and conducted initial literature review. C.M., R.G., L.O., A.M.S., N.S. and B.R.P. finalized the literature review and drafted the paper. C.L. provided critical data and technical supply chain input into the paper. C.M., R.G. and B.R.P. finalized, formatted and submitted the paper as per journal guidelines.

## Note

1 For the purpose of this article, the authors acknowledge the criteria that pertain to both formulations by mentioning both formulations, and mentioning one formulation when the criteria pertains to said formulation.
